# Spatial analysis of deaths from pulmonary tuberculosis in the city of São Luís, Brazil*

**DOI:** 10.1590/S1806-37132014000500011

**Published:** 2014

**Authors:** Marcelino Santos-Neto, Mellina Yamamura, Maria Concebida da Cunha Garcia, Marcela Paschoal Popolin, Tatiane Ramos dos Santos Silveira, Ricardo Alexandre Arcêncio

**Affiliations:** Nursing School, Federal University of Maranhão at Imperatriz, Imperatriz, Brazil; Program of Nursing in Public Health, University of São Paulo at Ribeirão Preto School of Nursing, Ribeirão Preto, Brazil; Program of Nursing in Public Health, University of São Paulo at Ribeirão Preto School of Nursing, Ribeirão Preto, Brazil; Program of Nursing in Public Health, University of São Paulo at Ribeirão Preto School of Nursing, Ribeirão Preto, Brazil; University of São Paulo at Ribeirão Preto School of Nursing, Ribeirão Preto, Brazil; Department of Maternal and Child Health and Public Health, University of São Paulo at Ribeirão Preto School of Nursing, Ribeirão Preto, Brazil

**Keywords:** Tuberculosis, pulmonary/mortality, Communicable disease control, Spatial analysis

## Abstract

**OBJECTIVE:**

To characterize deaths from pulmonary tuberculosis, according to sociodemographic and operational variables, in the city of São Luís, Brazil, and to describe their spatial distribution.

**METHODS:**

This was an exploratory ecological study based on secondary data from death certificates, obtained from the Brazilian Mortality Database, related to deaths from pulmonary tuberculosis. We included all deaths attributed to pulmonary tuberculosis that occurred in the urban area of São Luís between 2008 and 2012. We performed univariate and bivariate analyses of the sociodemographic and operational variables of the deaths investigated, as well as evaluating the spatial distribution of the events by kernel density estimation.

**RESULTS:**

During the study period, there were 193 deaths from pulmonary tuberculosis in São Luís. The median age of the affected individuals was 52 years. Of the 193 individuals who died, 142 (73.60%) were male, 133 (68.91%) were Mulatto, 102 (53.13%) were single, and 64 (33.16%) had completed middle school. There was a significant positive association between not having received medical care prior to death and an autopsy having been performed (p = 0.001). A thematic map by density of points showed that the spatial distribution of those deaths was heterogeneous and that the density was as high as 8.12 deaths/km^2^.

**CONCLUSIONS:**

The sociodemographic and operational characteristics of the deaths from pulmonary tuberculosis evaluated in this study, as well as the identification of priority areas for control and surveillance of the disease, could promote public health policies aimed at reducing health inequities, allowing the optimization of resources, as well as informing decisions regarding the selection of strategies and specific interventions targeting the most vulnerable populations.

## Introduction

The prevalence of and mortality from tuberculosis have declined worldwide, and most countries are likely to achieve the goal of reducing tuberculosis prevalence and mortality by 50% by 2015 in comparison with 1990 rates.^(^ 1 ^)^ The World Health Organization has launched a new challenge: the elimination of tuberculosis by 2050.^(^ 1 ^)^ Tuberculosis is the second leading cause of death from an infectious disease, accounting for approximately 1.3 million deaths worldwide in 2012; this demonstrates the severity of tuberculosis, especially in the 22 countries that collectively account for 80% of the disease burden.^(^ 2 ^)^

Brazil ranks 16th among the countries with the highest number of cases, the tuberculosis incidence and mortality rates in 2012 being 36.1/100,000 population and 2.4/100,000 population, respectively.^(^ 2 ^)^ Brazil has faced major challenges to achieving the ambitious goal of eliminating the disease. For example, in the city of São Luís, which is one of the priority cities for tuberculosis control in the country,^(^ 3 ^)^ the tuberculosis incidence and mortality rates in 2012 were 53.1/100,000 population and 3.9/100,000 population, respectively,^(^ 4 ^)^ indicating limited access to diagnosis and health care, as well as poor treatment adherence.^(^ 3 ^)^

Given that pulmonary tuberculosis (PTB) is transmissible, tuberculosis control measures should prioritize PTB over other clinical presentations. In addition, PTB is a preventable cause of death, meaning that deaths from PTB can be prevented by appropriate health promotion, protection, and recovery measures implemented in local health care systems.^(^ 5 ^)^

Studies of deaths from tuberculosis currently represent an important method for understanding the difficulties that health care systems face in controlling the disease and for identifying the most vulnerable groups. However, a literature review has shown that few studies have examined the spatial distribution of deaths from tuberculosis in the country.^(^ 6 ^)^

Although access to health care has improved in Brazil, an optimal level of equity has yet to be achieved, inequality resulting in health outcomes that are not always fair or acceptable,^(^ 7 ^)^ e.g., deaths from PTB. Therefore, the present study is evidently relevant, given that it can aid in strengthening health systems and services so that the problem of tuberculosis can be faced. 

The objectives of the present study were to characterize, on the basis of sociodemographic and operational variables, deaths from PTB in the city of São Luís in the 2008-2012 period; to determine whether there were differences between the PTB deaths that were confirmed by autopsy and those that were not in terms of the study variables; and to determine the geographic areas of the city in which PTB deaths were most common.

## Methods

**Figure 1:**
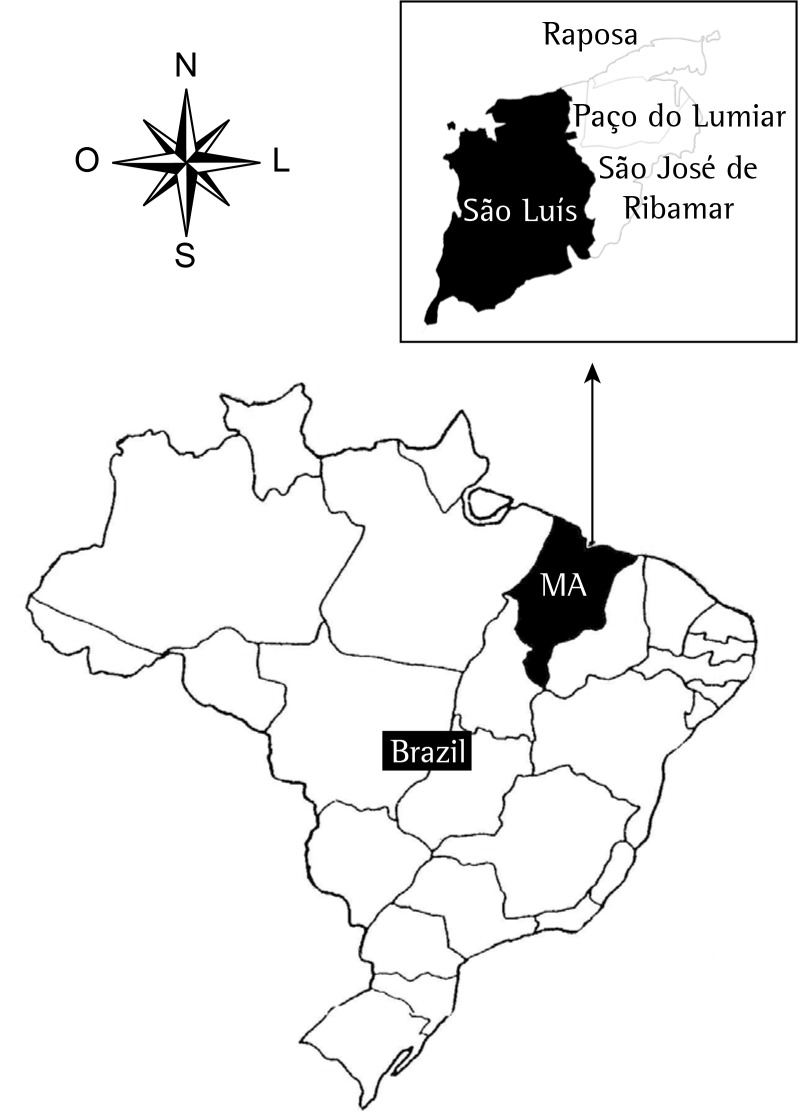
Map of Brazil showing the city of São Luís, in the state of Maranhão (MA). Adapted from Brazilian Institute of Geography and Statistics geographic databases.(8)

This was an exploratory ecological study conducted in the city of São Luís (Figure 1), which is located in northern Maranhão. The metropolitan area of São Luís comprises the municipalities of Paço do Lumiar, Raposa, and São José de Ribamar, among others. Specifically, the city of São Luís is located on the western portion of São Luís Island, between S02°28'21" W44°07'49" and S02°39'34" W44°20'59"; it has an area of 834.780 km^2^ and a population of 1,014,837 inhabitants.^(^ 8 ^)^

The study population consisted of individuals whose underlying cause of death was PTB (International Classification of Diseases, 10th revision-ICD-10-codes A15.0-A15.3 and A16.0-A16.2) and who resided in the urban area of São Luís between 2008 and 2012. 

Data were collected in July of 2013 from the *Sistema de Informação sobre Mortalidade* (SIM, Brazilian National Mortality Database) of the São Luís Municipal Department of Health Office for Epidemiological and Health Surveillance. Individuals who died in São Luís but resided elsewhere were excluded, as were those who died from clinical presentations other than PTB. 

The variables of interest were obtained from death certificates and included sociodemographic characteristics (including age, gender, skin color/ethnicity, marital status, level of education, and occupation) and operational variables (including place of death, medical care prior to death, autopsy, underlying cause of death, and physician completing the death certificate). 

After having analyzed the consistency of the data collected, we converted the data to STATISTICA, version 10.0 (StatSoft Inc., Tulsa, OK, USA), and the variables were regrouped and analyzed. Regarding the variable age, the individuals who died from PTB in São Luís were categorized on the basis of the median age, being therefore classified by age above or below the median. 

Using the same program, we subsequently performed a bivariate analysis cross-referencing the independent variables (sociodemographic and operational variables) with the dependent variable "death confirmed by autopsy" (yes, no). After that, we used the chi-square test for analysis of proportions. The probability of a type I error was set at 5%. At that point, death certificates that were incomplete or on which the cause of death was recorded as unknown were excluded from the study. 

In order to determine the areas in which deaths from PTB were most common, we used geocoding (with the freeware TerraView, version 4.2.2, developed by the Brazilian National Institute for Space Research), by standardizing the addresses of the individuals residing in the urban area of São Luís and comparing the addresses with a street segment digital map (StreetBase^(r)^; Imagem, São José dos Campos, Brazil) in World Geodetic System 1984 latitude and longitude, available in shapefile format. 

We subsequently performed kernel density estimation, which consists of an exploratory interpolation generating a density surface for visual identification of "hotspots"; the points within a region of influence are counted and then weighted by the distance between each point and the location of interest.^(^ 9 ^)^

A radius of 1,000 m being taken into consideration, a thematic map of the distribution of PTB deaths by home address was generated in ArcGIS software, version 10.2 (Esri, Redlands, CA, USA). 

The present study was approved by the Research Ethics Committee of the University of São Paulo at Ribeirão Preto School of Nursing (Ruling no. 259,935, May 8, 2013). 

## Results

We identified 221 deaths from tuberculosis. Of those, 193 were related to PTB. Of those, 190 (98.44%) were deaths of individuals whose death certificates listed PTB as the underlying cause of death, without mention of bacteriological or histological confirmation (ICD-10 16.2); 1 (0.52%) was the death of an individual whose death certificate read "pulmonary tuberculosis, no bacteriological or histological examination having been performed" (ICD-10 A16.1); 1 (0.52%) was the death of an individual whose death certificate read "pulmonary tuberculosis confirmed by histological examination" (ICD-10 A15.2); and 1 (0.52%) was the death of an individual whose death certificate read "pulmonary tuberculosis confirmed by sputum smear microscopy, with or without culture" (ICD-10 A15.0). 

The median age of the individuals who died in São Luís was 52 years, the youngest being 16 years old and the oldest being 93 years old. 

Table 1 shows the sociodemographic and operational characteristics of the individuals who died from PTB in São Luis, the results being presented in decreasing order of frequency. Most of the individuals who died were male (n = 142; 73.60%), Mulatto (n = 133; 68.91%), and single (n = 102; 53.13%). A greater proportion of the individuals had had 9 years of schooling (n = 64; 33.16%), and most were housekeepers (n = 79; 40.93%). With regard to the operational variables, most of the deaths occurred in a hospital (n = 143; 74.08%), and most of the individuals received medical care prior to death (n = 162; 83.94%, including those requiring hospitalization). Most of the deaths were certified by a substitute physician (n = 70; 36.27%). Most of the individuals who died did not undergo an autopsy (n = 108; 55.95%). Of the death certificates, 31 (16.05%) had no information as to whether an autopsy had been performed. 

**Table 1:** Sociodemographic and operational characteristics of individuals who died from pulmonary tuberculosis. São Luís, Brazil, 2008-2012.

Variable	n	%
Age		
≤ 52 years	98	50.78
> 52 years	95	49.22
Gender		
Male	142	73.60
Female	51	26.40
Skin color/ethnicity		
Brown (Mulatto)	133	68.91
White (Caucasian)	35	18.13
Black (African)	24	12.44
Yellow (Asian)	1	0.52
Marital status		
Single	102	53.13
Married	58	30.21
Widowed	21	10.94
Divorced	5	2.60
Steady partner	4	2.08
No data	2	1.04
Level of education		
9 years of schooling	64	33.16
High school	50	25.91
< 9 years of schooling	40	20.72
College (incomplete)	24	12.44
No schooling	9	4.66
College (complete)	4	2.08
No data	2	1.04
Occupation		
Housekeeper	79	40.93
Other	30	15.54
Rural worker	27	13.99
Homemaker	25	12.95
Pensioner	16	8.29
Unemployed	9	4.66
Student	5	2.60
No data	2	1.04
Place of death		
Hospital	143	74.08
Home	40	20.72
Street	7	3.64
Other	3	1.56
Medical care		
Yes	162	83.94
No	30	15.54
No data	1	0.52
Autopsy		
No	108	55.95
Yes	54	28.00
No data	31	16.05
Death certified by		
Substitute physician	70	36.27
Mortality Surveillance System	51	26.42
Attending physician	45	23.31
Other	24	12.44
Institute of Forensic Medicine	3	1.56
TOTAL	193	100.00
		

Table 2 shows the distribution of PTB deaths (an autopsy having been performed or not) according to the sociodemographic and operational characteristics. The variable skin color/ethnicity was found to be significantly associated with an autopsy having been performed (p = 0.003). An autopsy having been performed was also found to be significantly associated with death outside the hospital, e.g., at home or in the street (p = 0.001). Likewise, the variable "not having received medical care prior to death" was found to be significantly associated with an autopsy having been performed (p = 0.001). 

**Table 2:** Deaths from pulmonary tuberculosis (with or without autopsy confirmation), distributed according to sociodemographic and operational variables. São Luís, Brazil, 2008-2012.a

Variable	Autopsy^b^		p
	Yes	No	
Age			
≤ 52 years	23 (14.20)	58 (35.80)	0.182
> 52 years	31 (19.14)	50 (30.86)	
Gender			
Male	39 (24.07)	83 (51.23)	0.519
Female	15 (9.26)	25 (15.43)	
Skin color/ethnicity			
White (Caucasian)	4 (2.47)	27 (16.67)	0.003
Black (African)	3 (1.85)	17 (10.49)	
Yellow (Asian)	0 (0.00)	1 (0.62)	
Brown (Mulatto)	47 (29.01)	63 (38.89)	
Marital status^c^			
Single	31 (19.38)	50 (31.25)	0.708
Married	15 (9.38)	38 (23.75)	
Widowed	5 (3.13)	13 (8.13)	
Divorced	1 (0.63)	4 (2.50)	
Steady partner	1 (0.63)	2 (1.25)	
Level of education^c^			
No schooling	4 (2.50)	5 (3.13)	0.547
< 9 years of schooling	11 (6.88)	22 (13.75)	
9 years of schooling	19 (11.88)	34 (21.25)	
High school	16 (10.00)	29 (18.13)	
College (incomplete)	3 (1.88)	15 (9.38)	
College (complete)	0 (0.00)	2 (1.25)	
Occupation^c^			
Unemployed	3 (1.88)	6 (3.75)	0.227
Homemaker	11 (6.88)	8 (5.00)	
Rural worker	9 (5.63)	14 (8.75)	
Housekeeper	21 (13.13)	46 (28.75)	
Student	0 (0.00)	4 (2.50)	
Pensioner	0 (0.00)	13 (8.13)	
Other	9 (5.63)	16 (10.00)	
Place of death			
Hospital	13 (8.02)	102 (62.96)	0.001
Home	32 (19.75)	6 (3.70)	
Street	7 (4.32)	0 (0.00)	
Other	2 (1.23)	0 (0.00)	
Medical care			
Yes	29 (17.90)	105 (64.81)	0.001
No	25 (15.43)	3 (1.85)	
Underlying cause of death (ICD-10 code)			
15.0	0 (0.00)	1 (0.62)	0.675
15.2	0 (0.00)	1 (0.62)	
16.1	0 (0.00)	1 (0.62)	
16.2	54 (33.33)	105 (64.81)	
Death certified by			
Attending physician	1 (0.62)	28 (17.28)	0.001
Substitute physician	1 (0.62)	55 (33.95)	
Institute of Forensic Medicine	3 (1.85)	0 (0.00)	
Mortality Surveillance System	49 (30.25)	2 (1.23)	
Other	0 (0.00)	23 (14.20)	

ICD-10: International Classification of Diseases, 10th revision. aN = 162, except where otherwise indicated. bValues expressed as n (%). cN = 160

An autopsy having been performed was found to be significantly associated with death certified by the Mortality Surveillance System (p = 0.001). Most of the deaths after which an autopsy was not performed were certified by an attending physician or by a substitute physician (p = 0.001). 

During the study period, 183 deaths from PTB (95%) were geocoded. Of the cases that were not geocoded, 4 (2%) had incomplete addresses in the SIM and 2 (1%) lived in the rural area of São Luís. 

**Figure 2:**
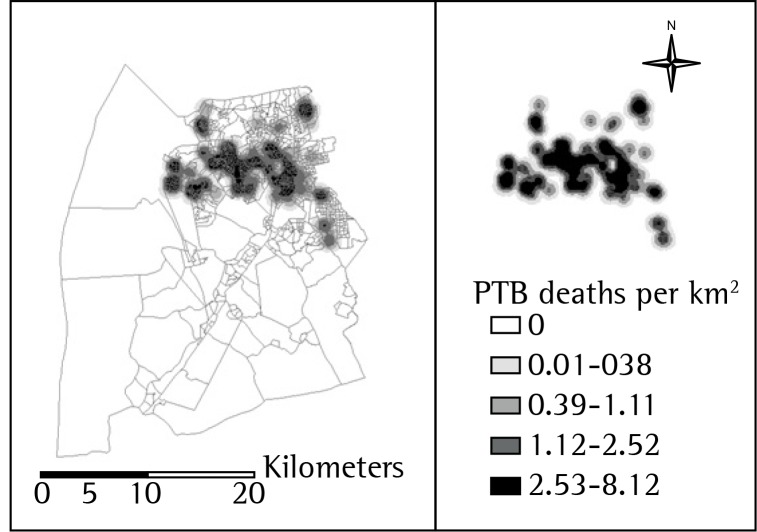
Dot density map of deaths from pulmonary tuberculosis (PTB) in the urban area of São Luís, Brazil, 2008-2012.

Figure 2 shows a thematic map of the distribution of PTB deaths in the urban area of São Luís by home address. The map highlights areas in which mortality rates were highest (deaths per km^2)^. These areas are represented by darker shades, denoting a heterogeneous spatial distribution of PTB deaths in São Luís during the study period. 

The hotspots for PTB deaths are concentrated in the districts of Anjo da Guarda, Liberdade, João de Deus, Bequimão, Cidade Operária, Coroadinho, Monte Castelo, and Centro (city center), the density ranging from 2.53 deaths/km^2^ to 8.12 deaths/km^2^. 

## Discussion

Although PTB is preventable, curable, and easily diagnosed-and although the Brazilian Unified Health Care System guarantees universal access to tuberculosis treatment, thus facilitating (at least to a certain extent) access to health services-4,500 people in Brazil die from the disease.^(^ 10 ^)^ The present study focused on deaths from PTB, which, as evidenced by the literature, is highly lethal and the main transmissible form of tuberculosis.^(^ 11 ^)^

The number of deaths from PTB in São Luís was relatively high in comparison with the number of deaths from PTB in other capitals of northeastern Brazil and the country as a whole.^(^ 4 ^)^ We found a significant number of deaths whose underlying cause was recorded as ICD-10 code 16.2, which is consistent with studies conducted in the city of Campo Grande^(^ 12 ^)^ and in the state of Rio de Janeiro, Brazil.^(^ 13 ^)^

This result indicates a major challenge for health care systems, i.e., a critical issue to be overcome; there is a possibility of false-positive results among the deaths whose cause was certified as PTB, given that most of the death certificates had no mention of bacteriological or histological confirmation. This might be due to the fact that the information was not recorded^(^ 10 ^)^ or to the fact that sputum smear microscopy is not prioritized in the hospital setting.^(^ 14 ^)^ This may have been a source of bias in the present study. 

With regard to the sociodemographic characteristics of the individuals who died from PTB in São Luís, the results of the present study are consistent with those of other studies,^(^ 13 ^-^ 23 ^)^ in which the proportion of deaths was found to be higher among males, a finding that corroborates reports that PTB is less common in females than in males. This might be due to the fact that males participate in the workforce more than do females and the fact that males use health services less than do females, as well as to the fact that the prevalence of HIV infection, alcoholism, and drug abuse is higher in males.^(^ 24 ^,^ 25 ^)^ However, these variables were not investigated in our study. 

The present study focused exclusively on deaths whose underlying cause was reported as being PTB, which is a preventable cause of death.^(^ 5 ^)^ Therefore, deaths associated with but not caused by tuberculosis were not included. 

With regard to age and marital status, deaths from PTB were found to be more common among individuals who were 52 years of age or younger and among those who were single, a finding that is consistent with those of studies conducted in the Brazilian states of Mato Grosso do Sul,^(^ 12 ^)^ Minas Gerais,^(^ 16 ^)^ Ceará,^(^ 25 ^)^ and Rio de Janeiro,^(^ 13 ^)^ as well as in Africa.^(^ 20 ^)^

With regard to skin color/ethnicity, the results of the present study are consistent with those of a study conducted in the city of Campo Grande^(^ 12 ^)^ and with those of a study conducted in other priority cities for tuberculosis control in the state of Mato Grosso do Sul,^(^ 11 ^)^ in which most of the deaths occurred in individuals who were Mulatto,^(^ 3 ^,^ 15 ^)^ but inconsistent with those of a study conducted in the city of São Paulo, Brazil,^(^ 18 ^)^ in which most of the deaths occurred in individuals who were White. 

With regard to the level of education and occupation, the findings of the present study are consistent with those of other studies,^(^ 11 ^,^ 12 ^,^ 16 ^,^ 18 ^)^ in which illiteracy or a low level of education and low-income jobs were reported as risk factors for PTB; all of the above are risk factors for PTB and are responsible for a higher incidence of the disease, as well as contributing to treatment nonadherence and death.^(^ 10 ^)^

With regard to operational characteristics, approximately 75% of the deaths analyzed in the present study occurred in a hospital. This is consistent with studies conducted in the Brazilian states of Rio de Janeiro^(^ 13 ^)^ and Mato Grosso do Sul,^(^ 11 ^)^ as well as with a study conducted in the city of São Paulo,^(^ 18 ^)^ all of which showed rates higher than 80%. The death of patients hospitalized for tuberculosis suggests that the primary health care system faces difficulties in management, in providing access to diagnostic resources, in case management, and in referring patients to other health services.^(^ 26 ^)^ Another possible explanation is treatment nonadherence, which predicts the development of multidrug-resistant tuberculosis and worsening of the disease; treatment discontinuation can lead to hospitalization when individuals seek health services.^(^ 20 ^)^

Analysis of the mortality rates for communicable diseases reflects the efficacy of prevention and control measures, as well as the quality of diagnosis and medical care, being of limited use when there is a high proportion of deaths without medical care or of deaths due to ill-defined causes.^(^ 5 ^)^ In the present study, approximately 15% of all deaths from PTB occurred without medical care, and, in most cases, no autopsy was performed. However, the records showed that 26% of the deaths were certified by the Mortality Surveillance System. 

Although an autopsy allows the diagnosis of diseases that were not suspected or elucidated before death, it should be used judiciously.^(^ 27 ^)^ In the present study, we found a significant association between not having received medical care prior to death and an autopsy having been performed. We also found that an autopsy was not performed in only 3 (1.85%) of the individuals who died without having received medical attention prior to death. 

The findings described above show the importance of a health care system that allows equity of access to health care, thus allowing tuberculosis cases to be diagnosed in a timely manner, especially in the primary health care setting, in order to prevent deaths. Autopsy confirmation of tuberculosis clearly shows the inadequacy of health care systems in reducing social inequalities in health.^(^ 28 ^)^

As is the case with other endemic diseases, tuberculosis is strongly influenced by the environment, and the disease has long been associated with a low socioeconomic status; therefore, there is a need to study the disease and combat it, its spatial distribution being taken into account.^(^ 19 ^)^

The spatial distribution of deaths from PTB (with emphasis on the heterogeneous distribution observed in the city of São Luís) should be considered the starting point of a process of investigation and surveillance that can lead to the identification of problem areas and failures of the health care system to provide health care for the target population. 

Our dot density map shows the sites where PTB deaths per km^2^ are most likely to occur, showing the spatial distribution of hotspots and revealing geographic inequalities related to events occurring in the city. This effectively contributed to the identification of geographic areas in which PTB deaths occurred and in which preventive/curative measures are therefore required in order to reorganize health services to meet the health needs of the population. 

The areas in which the density of deaths per km^2^ was highest coincided with areas in which housing conditions and home quality were classified as poor in a study conducted in São Luís.^(^ 29 ^)^ These areas include the following districts: Coroadinho; Ilhinha; Turu; Anjo da Guarda; Vila Nova; Vila Luizão; Vila Embratel; Liberdade; Sá Viana; Divineia; and parts of Cidade Operária. The aforementioned study^(^ 29 ^)^ showed that unplanned urban growth in São Luís resulted in subnormal areas, classified as lacking public services (most of which are essential). 

Curtis^(^ 28 ^)^ reported that health services tend to be ineffective and insufficient in areas in which housing and sanitation are poor, i.e., areas in which social inequality is high. Although we did not investigate the health care facilities in the aforementioned areas, we have reasons to assume that there is a relationship among the deaths from PTB, the areas in which those who died resided, and the health systems and services in those areas. 

In Brazil, high social inequality in access to health resources, education, income distribution, sanitation, and other aspects related to the standard of living of the population contributes to differences across social strata regarding the risk of illness^(^ 19 ^)^ and, consequently, the risk of death. 

As demonstrated in the present study, the identification of priority areas for tuberculosis control could promote public health policies aimed at reducing health inequalities, allowing the optimization of resources and teams for PTB control in the study setting, as well as informing decisions regarding the selection of strategies and specific interventions targeting the most vulnerable populations. 

For diseases for which reporting is mandatory, such as PTB, the use of data available in health information systems allows the monitoring of the problem, aids in the identification of relevant issues, and encourages the search for new interventions for disease control.^(^ 26 ^)^ Therefore, the data collected from the SIM allowed us to observe the dynamics and behavior of PTB in São Luís during the study period. 

As a source of information for the study of deaths in a given area, the SIM has limitations, including underreporting,^(^ 10 ^,^ 12 ^)^ which is one of the consequences of unequal access to health care. Another limitation is missing data on death certificates, complete records being important for health management and planning. 

The parameters used in the present study, particularly those used in the definition of the radius for kernel density estimation, were chosen on the basis of empirical knowledge and constitute one of the limitations of the present study. Nevertheless, we sought to choose parameters that contributed to the understanding of the study subject. 

Other limitations of the present study are due to its observational design, including interference from spurious variables or confounding factors. We should also take into consideration the ecological fallacy, which is the major limitation of ecological studies; therefore the results cannot be considered at the individual level. Finally, the use of secondary data constitutes yet another limitation of the present study; incomplete or missing data may have contributed to an information bias. 

The results of the present study can guide health managers and professionals in identifying priority areas for investment in health care, in order to eliminate preventable deaths from tuberculosis. The present study provides an opportunity to reconsider major clinical practice and environmental issues, as well as allowing us to reflect on the efficacy of public policies in reducing health inequalities and providing social protection to the population.
