# Supplementary material for: Spatial analysis of deaths from pulmonary tuberculosis in the city of São Luís, Brazil
Source: J Bras Pneumol. 2014 Sep-Oct;40(5):543–51. doi: 10.1590/S1806-37132014000500011 (PMC4263336; doi:10.1590/S1806-37132014000500011)
Supplement: Translation

# Translation: Análise espacial dos óbitos por tuberculose pulmonar em São Luís, Maranhão*

**Authors:** Marcelino Santos-Neto, Mellina Yamamura, Maria Concebida da Cunha Garcia, Marcela Paschoal Popolin, Tatiane Ramos dos Santos Silveira, Ricardo Alexandre Arcêncio

**Affiliations:** Curso de Enfermagem, Universidade Federal do Maranhão, Campus Imperatriz, Imperatriz (MA) Brasil; Programa Enfermagem em Saúde Pública, Escola de Enfermagem de Ribeirão Preto, Universidade de São Paulo, Ribeirão Preto (SP) Brasil; Programa Enfermagem em Saúde Pública, Escola de Enfermagem de Ribeirão Preto, Universidade de São Paulo, Ribeirão Preto (SP) Brasil; Programa Enfermagem em Saúde Pública, Escola de Enfermagem de Ribeirão Preto, Universidade de São Paulo, Ribeirão Preto (SP) Brasil; Escola de Enfermagem de Ribeirão Preto, Universidade de São Paulo, Ribeirão Preto (SP) Brasil; Departamento de Enfermagem Materno-Infantil e Saúde Pública, Escola de Enfermagem de Ribeirão Preto, Universidade de São Paulo, Ribeirão Preto (SP) Brasil

**Keywords:** Tuberculose pulmonar/mortalidade, Controle de doenças transmissíveis, Análise espacial

## Abstract

**OBJETIVO:**

Caracterizar os óbitos por tuberculose pulmonar em São Luís (MA) segundo variáveis sociodemográficas e operacionais e descrever sua distribuição espacial.

**MÉTODOS:**

Estudo ecológico e exploratório baseado em dados secundários oriundos das declarações de óbitos por tuberculose pulmonar do Sistema de Informação sobre Mortalidade. Foram incluídos todos os óbitos por tuberculose pulmonar ocorridos na zona urbana de São Luís entre 2008 e 2012. Foram realizadas análises univariadas e bivariadas das variáveis sociodemográficas e operacionais dos óbitos investigados e a distribuição espacial dos eventos por *kernel density estimation*.

**RESULTADOS:**

No período estudado, foram registrados 193 óbitos. A mediana de idade foi de 52 anos. Dos 193 indivíduos, 142 (73,60%) eram do sexo masculino, 133 (68,91%) da raça/cor parda, 102 (53,13%) eram solteiros, e 64 (33,16%) haviam completado o ensino fundamental. Observou-se que não ter recebido assistência médica antes do óbito teve uma associação estatisticamente significativa com a realização de necropsia (p = 0,001). O mapa temático por densidade de pontos demonstrou uma heterogeneidade na distribuição espacial dos óbitos, com taxas de até 8,12 óbitos/km^2^.

**CONCLUSÕES:**

As características sociodemográficas e operacionais dos óbitos por tuberculose pulmonar evidenciadas nessa investigação, bem como a identificação dos locais prioritários para o controle e a vigilância da doença, poderão auxiliar a gestão pública na diminuição das iniquidades em saúde e permitir uma otimização dos recursos, fornecendo subsídios para a escolha de estratégias e intervenções específicas direcionadas às populações mais vulneráveis.

## Introdução

A prevalência e a mortalidade por tuberculose vêm declinando em todo mundo, de modo que a maioria dos países provavelmente alcançará a meta de redução desses coeficientes em 50% em relação aos indicadores de 1990 até 2015.^(^ 1 ^)^ Um novo desafio já foi lançado pela Organização Mundial de Saúde para após o ano de 2015: a eliminação da tuberculose até 2050.^(^ 1 ^)^ A doença representa a segunda causa de óbitos entre as doenças infecciosas e causou, em 2012, cerca de 1,3 milhões de mortes no mundo, demonstrando sua gravidade, especialmente nos 22 países que concentram 80% da carga da doença.^(^ 2 ^)^

O Brasil está nesse grupo, ocupando a 16^a^ posição em número de casos, apresentando, em 2012, um coeficiente de incidência de 36,1/100.000 habitantes e uma taxa de mortalidade de 2,4/100.000 habitantes,^(^ 2 ^)^ e tem enfrentado grandes desafios para a consecução da ambiciosa meta de eliminação da doença. Por exemplo, São Luís (MA), um dos municípios prioritários para o controle da tuberculose no país,^(^ 3 ^)^ apresentou, naquele mesmo ano, um coeficiente de incidência de 53,1/100.000 habitantes e uma taxa de mortalidade de 3,9/100.000 habitantes,^(^ 4 ^)^ evidenciando dificuldades no acesso ao sistema de saúde e ao diagnóstico, assim como na adesão ao tratamento.^(^ 3 ^)^

Dentre as formas clínicas da doença, a tuberculose pulmonar (TBP) deve ser priorizada em relação às ações de controle por se tratar da forma clínica transmissível. Ademais, a TBP faz parte da lista de causas de mortes evitáveis, haja vista que, se instituídas ações adequadas de promoção, proteção e recuperação da saúde dos indivíduos e famílias pelos sistemas locais de saúde, esses eventos não ocorreriam.^(^ 5 ^)^

Baseado nesse contexto, estudos sobre óbitos por tuberculose representam atualmente um importante investimento para decifrar os nós críticos dos sistemas de saúde no controle da doença e também para realçar os grupos mais vulneráveis ao fenômeno. No entanto, após uma revisão da literatura, constataram-se poucos estudos que se propusessem analisar a distribuição espacial dos óbitos por tuberculose no país.^(^ 6 ^)^

Cabe mencionar que mesmo diante dos avanços que ocorreram no Brasil em termos de melhoria do acesso aos serviços de saúde, esses ainda não alcançaram um nível ideal de equidade, o que tem repercutido em resultados sanitários nem sempre justos ou aceitáveis,^(^ 7 ^)^ como os óbitos por TBP. Assim, fica evidente a relevância do presente estudo, visto que esse poderá fornecer elementos para o fortalecimento dos sistemas e serviços de saúde no enfrentamento da problemática da tuberculose.

Diante do exposto, o presente estudo teve como objetivos caracterizar os óbitos por TBP ocorridos em São Luís (MA) no período entre 2008 e 2012 segundo variáveis sociodemográficas e operacionais, verificar se houve diferenças entre os óbitos confirmados por necropsia (ou não) no tocante às variáveis investigadas e, por fim, estimar as áreas geográficas do município mais vulneráveis a ocorrência desses óbitos.

## Métodos

Trata-se de um estudo ecológico e exploratório, realizado no município de São Luís, Brasil (Figura 1), localizado ao norte do Estado do Maranhão, onde também estão os municípios de Paço do Lumiar, Raposa e São José de Ribamar, constituintes da região metropolitana de São Luís. O município ocupa especificamente a porção ocidental da Ilha de São Luís, nas coordenadas geográficas 02°28'21" e 02°39'34", latitude sul, e 44°07'49" e 44°20'59", longitude oeste; apresenta uma área de 834,780 km^2^ e uma população de 1.014.837 habitantes.^(^ 8 ^)^

**Figura 1:** Mapa do Brasil com destaque para a cidade de São Luís, estado do Maranhão (MA). Adaptado de bases geográficas, Instituto Brasileiro de Geografia e Estatística.(8

A população do estudo foi composta de casos que evoluíram para óbito tendo como causa básica TBP - Classificação Internacional de Doenças, 10ª revisão (CID-10), correspondente a A15.0-A15.3 e A16.0-A16.2 - na população residente da zona urbana do município entre os anos de 2008 e 2012.

Os dados foram coletados em julho de 2013 junto ao Sistema de Informação sobre Mortalidade (SIM) da Superintendência de Vigilância Epidemiológica e Sanitária da Secretaria Municipal de Saúde. Foram excluídos os óbitos, ocorridos no município de São Luís, de indivíduos residentes de outras localidades e óbitos por tuberculose apresentando outra forma clínica que não a pulmonar.

As variáveis de interesse do presente estudo foram obtidas da declaração de óbito utilizada no Brasil e compreenderam características sociodemográficas, como idade, sexo, raça/cor, estado civil, escolaridade e ocupação, além de variáveis operacionais, tais como local de ocorrência do óbito, assistência médica, necropsia, causa básica e médico responsável pelo atestado de óbito.

Após a análise de consistência de dados coletados, esses foram convertidos para uso com o programa STATISTICA, versão 10.0 (StatSoft Inc., Tulsa, OK, EUA), procedendo-se a recategorização das variáveis e a sua análise. Em relação à variável idade, os indivíduos que evoluíram para óbito foram categorizados a partir da mediana da idade, sendo classificados, portanto, por idade acima ou abaixo do valor obtido.

Posteriormente, efetuou-se, utilizando o mesmo programa, realizou-se uma análise bivariada com cruzamento das variáveis independentes (variáveis sociodemográficas e operacionais) com a variável dependente "confirmação do óbito por necropsia" (sim, não), aplicando-se então o teste do qui-quadrado para a análise de proporções. Fixou-se a probabilidade de erro tipo I em 5%. Nessa fase das análises, os registros de óbitos denominados ignorados ou não preenchidos foram eliminados.

Para estimar as áreas mais vulneráveis aos óbitos por TBP, inicialmente procedeu-se a geocodificação utilizando o programa TerraView, versão 4.2.2 (desenvolvido no Instituto Nacional de Pesquisas Espaciais e de acesso público), a partir da padronização e equiparação dos endereços dos casos residentes da zona urbana de São Luís com um mapa digital de segmento de logradouros StreetBase^(r)^ (Imagem, São José dos Campos, Brasil) em projeção *Latlong/WGS84*, disponibilizado na extensão *shapefile*. 

Posteriormente, recorreu-se à técnica de análise da densidade de pontos, definida como *kernel density estimation*, que consiste na interpolação exploratória que gera uma superfície de densidade para a identificação visual de "áreas quentes", uma vez que realiza a contagem de pontos dentro de uma região de influência, sendo ponderados pela distância de cada um à localização de interesse.^(^ 9 ^)^

Considerando raio de 1.000 m, o mapa temático da distribuição da densidade dos óbitos por TBP, segundo o endereço de residência, foi gerado no programa ArcGIS, versão 10.2 (Esri, Redlands, CA, EUA).

O estudo foi aprovado pelo Comitê de Ética da Escola de Enfermagem de Ribeirão Preto da Universidade de São Paulo, sob o parecer número 259.935, emitido em 8 de maio de 2013.

## Resultados

Foram identificados 221 óbitos por tuberculose, dos quais 193 estavam relacionados à TBP. Desses, 190 (98,44%) apresentavam o registro como causa básica do óbito por TBP, sem menção de confirmação bacteriológica ou histológica (CID-10 16.2); 1 (0,52%) com o registro "tuberculose pulmonar, sem realização de exame bacteriológico ou histológico" (CID-10 A16.1); 1 (0,52%) com o registro "tuberculose pulmonar com confirmação histológica" (CID-10 A15.2); e 1 (0,52%) com o registro "tuberculose pulmonar, com confirmação por exame microscópico da expectoração, com ou sem cultura" (CID-10 A15.0).

A mediana de idade dos indivíduos que morreram foi de 52 anos, tendo o caso mais novo a idade de 16 anos e o mais velho, de 93 anos. 

A Tabela 1 apresenta as características sociodemográficas e operacionais dos indivíduos que foram a óbito por TBP em São Luis, cujos resultados foram ordenados de forma decrescente de frequência. A maioria dos óbitos ocorreu em indivíduos do sexo masculino (n = 142; 73,60%), de raça/cor parda (n = 133; 68,91%) e solteiros (n = 102; 53,13%). Ainda, em relação à escolaridade, uma maior proporção tinha o ensino fundamental completo (n = 64; 33,16%), enquanto a ocupação mais frequente foi a de serviços gerais (n = 79; 40,93%). Com relação aos indicadores operacionais, observou-se que a maior proporção dos óbitos ocorreu em um hospital (n = 143; 74,08%) e que a maioria dos pacientes recebeu assistência médica antes do óbito (n = 162; 83,94%), incluindo, nesses números, os casos que demandaram hospitalização. Quanto ao responsável pelos registros, a maior parcela foi realizada por um médico substituto (n = 70; 36,27%). Ademais, verificou-se que a maior proporção dos indivíduos que foram a óbito não foi submetidas à necropsia (n = 108; 55,95%), enquanto 31 registros (16,05%) não apresentavam esse tipo de informação.

**Tabela 1:** Características sociodemográficas e operacionais dos óbitos por tuberculose pulmonar. São Luís (MA), Brasil, 2008-2012.

Variáveis	n	%
Idade		
≤ 52 anos	98	50,78
> 52 anos	95	49,22
Sexo		
Masculino	142	73,60
Feminino	51	26,40
Raça/cor		
Parda	133	68,91
Branca	35	18,13
Preta	24	12,44
Amarela	1	0,52
Estado civil		
Solteiro	102	53,13
Casado	58	30,21
Viúvo	21	10,94
Divorciado	5	2,60
União estável	4	2,08
Ignorado	2	1,04
Escolaridade		
Ensino fundamental completo	64	33,16
Ensino médio	50	25,91
Ensino fundamental incompleto	40	20,72
Ensino superior incompleto	24	12,44
Sem escolaridade	9	4,66
Ensino superior completo	4	2,08
Ignorado	2	1,04
Ocupação		
Serviços gerais	79	40,93
Outros	30	15,54
Trabalhador rural e correlatos	27	13,99
Dona de casa	25	12,95
Pensionista	16	8,29
Desempregado	9	4,66
Estudante	5	2,60
Ignorado	2	1,04
Local de ocorrência do óbito		
Hospital	143	74,08
Domicílio	40	20,72
Via pública	7	3,64
Outros	3	1,56
Assistência médica		
Sim	162	83,94
Não	30	15,54
Ignorado	1	0,52
Necropsia		
Não	108	55,95
Sim	54	28,00
Ignorado	31	16,05
Óbito atestado por médico		
Substituto	70	36,27
Serviço de Vigilância de Óbito	51	26,42
Assistente	45	23,31
Outro	24	12,44
Instituto Médico Legal	3	1,56
TOTAL	193	100,00
		

A Tabela 2 apresenta os óbitos por TBP, submetidos ou não a necropsia, segundo as características sociodemográficas e operacionais. A variável raça/cor teve uma associação estatisticamente significativa com a realização da necropsia (p = 0,003). Também apresentou uma associação estatisticamente significativa a realização da necropsia com a ocorrência do óbito fora do ambiente hospitalar, como no domicílio, na via pública, entre outros (p = 0,001). Da mesma forma, a variável "não ter recebido assistência médica" apresentou uma associação estatisticamente significativa com a realização da necropsia (p = 0,001).

**Tabela 2:** Óbitos por tuberculose pulmonar, submetidos ou não a necropsia, segundo variáveis sociodemográficas e operacionais. São Luís (MA), Brasil, 2008-2012.a

Variáveis	Necropsia^b^		p
	Sim	Não	
Idade			
≤ 52 anos	23 (14,20)	58 (35,80)	0,182
> 52 anos	31 (19,14)	50 (30,86)	
Sexo			
Masculino	39 (24,07)	83 (51,23)	0,519
Feminino	15 (9,26)	25 (15,43)	
Raça/cor			
Branca	4 (2,47)	27 (16,67)	0,003
Preta	3 (1,85)	17 (10,49)	
Amarela	0 (0,00)	1 (0,62)	
Parda	47 (29,01)	63 (38,89)	
Estado civil^c^			
Solteiro	31 (19,38)	50 (31,25)	0,708
Casado	15 (9,38)	38 (23,75)	
Viúvo	5 (3,13)	13 (8,13)	
Divorciado	1 (0,63)	4 (2,50)	
União estável	1 (0,63)	2 (1,25)	
Escolaridade^c^			
Sem escolaridade	4 (2,50)	5 (3,13)	0,547
Ensino fundamental incompleto	11 (6,88)	22 (13,75)	
Ensino fundamental completo	19 (11,88)	34 (21,25)	
Ensino médio	16 (10,00)	29 (18,13)	
Ensino superior incompleto	3 (1,88)	15 (9,38)	
Ensino superior completo	0 (0,00)	2 (1,25)	
Ocupação^c^			
Desempregado	3 (1,88)	6 (3,75)	0,227
Dona de casa	11 (6,88)	8 (5,00)	
Trabalhador rural e correlatos	9 (5,63)	14 (8,75)	
Serviços gerais	21 (13,13)	46 (28,75)	
Estudante	0 (0,00)	4 (2,50)	
Pensionista	0 (0,00)	13 (8,13)	
Outros	9 (5,63)	16 (10,00)	
Local de ocorrência do óbito			
Hospital	13 (8,02)	102 (62,96)	0,001
Domicílio	32 (19,75)	6 (3,70)	
Via pública	7 (4,32)	0 (0,00)	
Outros	2 (1,23)	0 (0,00)	
Assistência médica			
Sim	29 (17,90)	105 (64,81)	0,001
Não	25 (15,43)	3 (1,85)	
Causa básica (código CID-10)			
15.0	0 (0,00)	1 (0,62)	0,675
15.2	0 (0,00)	1 (0,62)	
16.1	0 (0,00)	1 (0,62)	
16.2	54 (33,33)	105 (64,81)	
Óbito atestado por médico			
Assistente	1 (0,62)	28 (17,28)	0,001
Substituto	1 (0,62)	55 (33,95)	
Instituto Médico Legal	3 (1,85)	0 (0,00)	
Serviço de Vigilância de Óbito	49 (30,25)	2 (1,23)	
Outro	0 (0,00)	23 (14,20)	

CID-10: Classificação Internacional de Doenças, 10ª revisão

a N = 162, exceto onde indicado

b Valores expressos em n (%)

c N = 160.

Verificou-se ainda uma associação estatisticamente significativa entre a realização da necropsia e óbito atestado pelo Serviço de Vigilância de Óbito (p= 0,001). Já entre os óbitos que não foram submetidos à necropsia, observou-se que sua maior proporção foi atestada por um médico assistente ou substituto (p = 0,001).

Foram geocodificados 183 óbitos por TBP (95%) no período definido para a investigação. Entre os casos não geocodificados, 4 (2%) apresentavam endereçamentos incompletos no SIM e 2 (1%) residiam na zona rural do município.

O mapa temático da densidade de pontos dos óbitos por TBP ocorridos na zona urbana do município investigado, segundo o endereço de residência, está apresentado na Figura 2. Nesse mapa destacam-se áreas com taxas de óbitos mais elevadas (óbitos por km^2)^, representadas por tons mais escuros, denotando uma heterogeneidade na distribuição espacial dos óbitos no município no período estudado.

**Figura 2:** Mapa da distribuição da densidade de pontos dos óbitos por tuberculose pulmonar (TBP) ocorridos na zona urbana de São Luís (MA), 2008-2012.

Observa-se que as áreas quentes de ocorrência dos óbitos por TBP estão concentradas nos bairros Anjo da Guarda, Liberdade, João de Deus, Bequimão, Cidade Operária, Coroadinho, Monte Castelo e Centro, com uma variação de 2,53 a 8,12 óbitos por TBP por km^2^.

## Discussão

Embora a TBP seja evitável, curável, de fácil diagnóstico e cujo tratamento de cobertura universal é oferecido pelo Sistema Único de Saúde, o que de certo modo favoreceria o acesso aos serviços de saúde, 4,5 mil pessoas ainda morrem no país em decorrência da doença.^(^ 10 ^)^ Ressalta-se que os óbitos identificados no presente estudo referem-se à forma clínica pulmonar, que conforme evidencia a literatura, é de elevada letalidade e representa a principal forma transmissível da doença.^(^ 11 ^)^

O número de óbitos por TBP ocorridos em São Luís foi relativamente alto quando comparado com as demais capitais do nordeste brasileiro e do país como um todo.^(^ 4 ^)^ Observou-se inicialmente, no cenário em questão, um número expressivo de óbitos que foram registrados com o código CID-10 16.2 como causa básica, tal como em estudos realizados em Campo Grande (MS)^(12) ^e no estado do Rio de Janeiro.^(^ 13 ^)^

Esse resultado remete a uma importante questão problemática para os sistemas de saúde e, assim, a um nó crítico a ser superado por eles, haja vista a possibilidade da existência de resultados falso-positivos entre os óbitos que tiveram como causa declarada a TBP, uma vez que a maioria dos registros não apresentava a menção de confirmação bacteriológica ou histológica. Dentre as hipóteses explicativas para esse achado está a ausência de registro dessa informação^(^ 10 ^)^ e também a não priorização da baciloscopia do escarro no cenário hospitalar.^(^ 14 ^)^ Esses resultados podem ter gerado algum viés à investigação.

Analisando-se as características sociodemográficas dos indivíduos que evoluíram para óbito, foi possível evidenciar que os resultados do presente estudo coadunam-se com os de outros estudos^(^ 13 ^-^ 23 ^)^ que relatam que a maior proporção dos óbitos ocorre na população masculina, reiterando, portanto, relatos de que a TBP é menos comum nas mulheres do que nos homens. Possivelmente, atribui-se ao fato de os homens estarem mais presentes no mercado de trabalho e menos presentes nos serviços de saúde, além de apresentarem maiores prevalências de infecção pelo HIV, de alcoolismo e de uso abusivo de drogas,^(^ 24 ^,^ 25 ^)^ embora essas variáveis não tenham sido exploradas em nossa casuística.

Vale reiterar que, na presente investigação, foram considerados somente os óbitos que referiam a TBP como causa principal, haja vista que ela faz parte da lista de causas de mortes tidas como evitáveis.^(^ 5 ^)^ Em face dessa justificativa teórica, os casos em que a tuberculose estava apenas associada ao óbito não foram incluídos.

No que diz respeito à idade e ao estado civil, evidenciou-se que os óbitos por TBP ocorreram em maior quantidade entre pessoas com idade ≤ 52 anos e solteiros, corroborando resultados de estudos realizados no Brasil em cidades dos estados de Mato Grosso do Sul,^(^ 12 ^)^ Minas Gerais,^(^ 16 ^)^ Ceará^(^ 25 ^)^ e Rio de Janeiro^(13) ^e, ainda, na África.^(^ 20 ^)^

Quanto à raça/cor, os resultados obtidos expressaram equivalência ao estudo realizado no município de Campo Grande (MS)^(^ 12 ^)^ e em demais municípios prioritários daquele estado^(^ 11 ^)^ para o controle da tuberculose, denotando que a maior parte dos óbitos ocorreu em indivíduos de raça/cor parda,^(^ 3 ^,^ 15 ^)^ mas diferindo de um estudo realizado no município de São Paulo,^(^ 18 ^)^ onde a raça/cor branca foi a prevalente.

Os dados de escolaridade e ocupação encontrados no presente estudo foram concordantes com os de alguns estudos,^(11,12,16,18) ^que relatam o analfabetismo ou a baixa escolaridade e a ocupação em situações menos privilegiadas do ponto de vista financeiro como fatores de risco para a TBP, reflexos de um conjunto de condições socioeconômicas precárias que aumentam a vulnerabilidade à TBP e são responsáveis pela maior incidência dessa enfermidade, além de contribuírem para a não adesão ao tratamento e para o aumento da taxa de abandono do tratamento, bem como para a ocorrência de óbito.^(^ 10 ^)^

No tocante às características operacionais, aproximadamente 75% dos óbitos analisados ocorreram em um hospital, situação semelhante evidenciada em estudos nacionais realizados nos estados de Rio de Janeiro^(13) ^e Mato Grosso do Sul,^(^ 11 ^)^ assim como no município de São Paulo,^(^ 18 ^)^ com registros superiores a 80%. A evolução para o óbito de pacientes hospitalizados por tuberculose sugere a dificuldade da Atenção Primária à Saúde na gestão, na oferta de recursos diagnósticos, no manejo dos casos e no sistema de transferência a outros serviços de saúde.^(^ 26 ^)^ Há também outro fator que ajuda a explicar esse resultado, que se refere ao abandono do tratamento, sendo esse um preditor para o desenvolvimento de tuberculose resistente a múltiplas drogas e para o agravamento da doença; o tratamento interrompido pode levar à hospitalização quando os indivíduos procuram os serviços de saúde.^(^ 20 ^)^

A análise das taxas de mortalidade por doenças transmissíveis reflete a efetividade de medidas de prevenção e controle, bem como as condições de diagnóstico e de assistência médica dispensada, e apresenta uma restrição de uso sempre que ocorre uma elevada proporção de óbitos sem assistência médica ou por causas mal definidas.^(^ 5 ^)^ Na presente investigação, evidenciou-se que aproximadamente 15% dos óbitos por TBP ocorreram sem assistência médica e, na maioria dos casos, a necropsia não foi realizada. Entretanto, segundo os registros, 26% dos óbitos foram atestados pelo Serviço de Vigilância de Óbitos.

Destaca-se que a necropsia possibilita o diagnóstico de doenças que não foram suspeitas ou elucidadas antes da morte; no entanto, o seu uso deve ser criterioso.^(^ 27 ^)^ Os achados da pesquisa revelam números relevantes nesse sentido, devido à associação significativa entre as variáveis "não ter recebido assistência médica antes do óbito" e "ter sido submetido à necropsia". Verificou-se, ainda, que somente 3 casos de óbito (1,85%) que não receberam atenção médica previamente ao óbito tampouco não foram submetidos à necropsia.

A discussão que sucede a esse resultado é da importância de um sistema de saúde que permita a equidade do acesso aos serviços de saúde e, dessa forma, permita que casos de tuberculose sejam diagnosticados em tempo oportuno, especialmente na Atenção Primária à Saúde a fim de evitar a ocorrência de óbitos. A confirmação da tuberculose numa necropsia exprime exatamente a fragilidade e a insuficiência dos sistemas de serviços de saúde na redução das iniquidades sociais em saúde.^(^ 28 ^)^

O comportamento da tuberculose, como o de outras endemias, é fortemente influenciado pelo meio, sendo possível evidenciar que a associação entre tuberculose e precárias condições socioeconômicas data dos primórdios da epidemiologia dessa doença, reiterando, portanto, a necessidade de estudá-la e sobre ela intervir, levando-se em consideração sua distribuição espacial.^(^ 19 ^)^

Nessa linha de raciocínio, a distribuição espacial dos óbitos por TBP, com ênfase na distribuição heterogênea observada na cidade de São Luís, deve ser considerada como o momento inicial de um processo de investigação e vigilância, que pode desencadear a focalização de áreas-problema e a identificação de elos frágeis do sistema de atenção à saúde da população alvo.

O mapa da densidade de pontos aponta visualmente as localidades mais vulneráveis à ocorrência de óbitos por TBP por km^2^, indicando espacialmente as áreas quentes, e permite evidenciar desigualdades de eventos na área geográfica do município. Essa visualização contribuiu efetivamente na identificação de áreas geográficas nas quais ocorreram óbitos por TBP e que necessitam, portanto, de maior atenção, seja preventiva, seja curativa, tendo como propósito a reorganização dos serviços de saúde para responder às necessidades de saúde da população.

As áreas com maior densidade de óbitos por km^2 ^também coincidem com as áreas classificadas como precárias quanto às condições de habitação e qualidade dos domicílios, conforme evidenciou um estudo realizado na capital maranhense.^(^ 29 ^)^ Nessas áreas estão os bairros do Coroadinho, Ilhinha, Turu, Anjo da Guarda, Vila Nova, Vila Luizão, Vila Embratel, Liberdade, Sá Viana, Divineia e áreas da Cidade Operária. Aquele estudo^(^ 29 ^)^ destacou ainda que o crescimento urbano de São Luís, sem um planejamento prévio, resultou em áreas consideradas subnormais, classificadas como carentes de serviços públicos e essenciais em sua maioria.

Curtis^(28) ^tem apontado que áreas com grandes privações em relação à moradia digna e saneamento básico e, portanto, com maior iniquidade social, tendem também a experimentar serviços de saúde pouco resolutivos e com limitações em termos de oferta e de elenco de serviços. Embora a rede de serviços de saúde nas referidas áreas não tenham sido investigadas, há razões que fazem os autores supor a relação entre os óbitos, as áreas onde os casos residiam e os sistemas e serviços de saúde nelas presentes.

A acentuada desigualdade social no Brasil, observada no acesso aos recursos de saúde, na educação, na distribuição de renda, no saneamento básico, na educação e em outros constituintes do padrão de vida da população, favorecem divergências relacionadas ao risco de adoecer^(19) ^e, consequentemente, de evoluir a óbito nos diversos estratos sociais.

Ressalta-se ainda que o conhecimento de locais prioritários para o controle, como demonstrado pelo presente estudo, poderá auxiliar a gestão pública na diminuição das iniquidades em saúde e permitir uma otimização dos recursos e das equipes no controle da TBP no cenário em questão, fornecendo subsídios para a escolha de estratégias e para intervenções específicas direcionadas às populações mais vulneráveis.

Em se tratando dos agravos de notificação compulsória, em destaque a TBP, a utilização de dados disponíveis no sistema de informação em saúde permite o acompanhamento do problema, colaborando para identificação de aspectos relevantes e incentivando a busca de novas intervenções para o controle da doença.^(^ 26 ^)^ Assim, os dados coletados junto ao SIM permitiram observar a dinâmica e o comportamento da TBP na capital do estado do Maranhão durante o período estudado.

Cabe mencionar que o SIM, como fonte de informações para o estudo dos óbitos de uma determinada região, tem suas fragilidades; uma delas é a própria subnotificação no país,^(^ 10 ^,^ 12 ^)^ que é uma das consequências da iniquidade do acesso aos serviços de saúde. Destacam-se também como fragilidade as lacunas no preenchimento dos registros, que são informações relevantes à gestão e ao planejamento em saúde.

Dentre as limitações da investigação, os parâmetros adotados pelos autores, especialmente na definição do raio para a estimativa de densidade *kernel*, são de responsabilidade dos autores, com base no conhecimento empírico. Houve uma grande preocupação dos mesmos em escolher parâmetros que pudessem oferecer maiores contribuições para a compreensão do objeto investigado.

Ademais, como estudo observacional, o presente estudo tem limitações concernentes ao tipo de estudo, como a interferência de variáveis espúrias ou fatores de confusão. Há que se destacar também a maior limitação de estudos ecológicos, que é a falácia ecológica; portanto, os resultados não podem ser considerados em nível individual. Acrescenta-se, por último, o próprio uso de dados secundários, cujos dados incompletos ou ignorados podem ter trazido um viés de informação à investigação.

Os resultados do presente estudo poderão orientar gestores e profissionais de saúde quanto às áreas prioritárias de investimento em saúde e, assim, eliminar a ocorrência de óbitos evitáveis e injustos por tuberculose. O presente estudo traz aspectos importantes a serem repensados tanto do ponto de vista da prática clínica, quanto também do ambiente, e faz refletir ainda acerca da efetividade das políticas públicas na redução das iniquidades em saúde e na proteção social da população.
